# Diagnostic accuracy of a single‐lead portable ECG device for measuring QTc prolongation

**DOI:** 10.1111/anec.12683

**Published:** 2019-07-26

**Authors:** Charlotte L. Bekker, Fauve Noordergraaf, Steven Teerenstra, Gheorghe Pop, Bart J. F. van den Bemt

**Affiliations:** ^1^ Department of Pharmacy, Radboud University Medical Center Radboud Institute for Health Sciences Nijmegen The Netherlands; ^2^ Department of Cardiology Radboud University Medical Center Radboud Institute for Health Sciences Nijmegen The Netherlands; ^3^ Group Biostatistics, Department for Health Evidence Radboud University Medical Center Radboud Institute for Health Sciences Nijmegen The Netherlands; ^4^ Department of Pharmacy Sint Maartenskliniek Nijmegen The Netherlands

**Keywords:** cardiac monitoring, electrocardiography, portable device, QTc, QT‐interval

## Abstract

**Background:**

To assess the diagnostic accuracy of a single‐lead portable ECG device for measuring QTc‐intervals in comparison with a standard 12‐lead ECG.

**Methods:**

Adult patients visiting the cardiology outpatient clinic for a 12‐lead recording were also measured with a portable single‐lead ECG recorder (HeartcheckTM). QTc‐intervals were determined by two cardiologists. Perfect agreement was defined as a limit of ≤10 ms between the two measurement methods.

**Results:**

Hundred one ECGs were recorded. QTc‐interval mean differences between the two measurement methods was substantially outside our definition of perfect agreement (‐31.9 [SD±41.3] ms).

**Conclusion:**

In conclusion, the Heartcheck single‐lead ECG device is not accurate for measuring QTc‐intervals.

## 1 INTRODUCTION

QTc‐interval prolongation of the heart may lead to potential fatal cardiac arrhythmias, such as Torsades des Pointes (Al‐Khatib, LaPointe, Kramer, & Califf, 2003). Multiple risk factors are associated with QTc prolongation, including congenital QTc prolongation, female gender, age ≥65 years, ischemic heart disease, electrolyte disturbances, and liver/kidney failure (Vandael, Vandenberk, Vandenberghe, Willems, & Foulon, 2017). Furthermore, over 170 drugs have the potential to prolong the QTc interval, such as antiarrhythmic drugs, antipsychotics, and antimycotics. The incidence of fatal arrhythmias as a result of QTc‐prolongation is relatively rare and not exactly known. However, QTc‐prolonging drugs are frequently prescribed and potentially many patients are at risk.

To provide prevention of fatal arrhythmias due to QTc‐prolongation, 12‐lead electrocardiogram (ECG) monitoring of the QTc interval in patients taking these drugs is recommended. However, ECGs are rarely recorded due to limited awareness among physicians regarding QTc‐interval monitoring and low feasibility to record an ECG in all these patients (Warnier et al., 2015).

Over the past years, single‐lead easy to use portable ECG devices have become available. Such devices have been proven to be accurate for atrial fibrillation screening compared with the gold‐standard 12‐lead ECG (Haberman et al., 2015; Lau et al., 2013; Torfs et al., 2014) and have been used already for this purpose in clinical practice (Narasimha et al., 2018). Currently, little is known about the diagnostic accuracy of such devices for QTc‐prolongation. Studies that assessed the accuracy of portable ECG devices for QTc prolongation included a small number of patients (*n* = 5) (Chung & Guise, 2015) or included a device that is less easy to use in primary care (Garabelli et al., 2016). Therefore, this study aims to assess the diagnostic accuracy of a single‐lead portable ECG device for measuring QTc intervals in comparison with a standard 12‐lead ECG in a large group of patients visiting a cardiology outpatient clinic.

## 2 METHODS

Consecutive adult (≥18 years) patients visiting the outpatient cardiology department of the Radboud University Medical Center with an indication for a 12‐lead ECG recording in February 2018 were included after oral consent was obtained. Patients with the presence of a pacemaker or implantable defibrillator were excluded. The study was reviewed by the Medical Research and Ethics committee of the Radboud University Medical Center (protocol reference number 2018‐4068), which decided that the Medical Research Involving Human Subjects Act (WMO) was not applicable to this study.

Immediately after the standard 12‐lead ECG (lying position), an ECG recording was taken with the (Heartcheck^TM^; Canada) device (CE class II certified, code 0123), which is a lightweight, portable single‐lead ECG recording system with a digital computer software system for analysis. It records in 30 s an ECG, which is taken by placing two electrodes on the patient's left wrist and one on the right wrist.

A cardiologist in training interpreted the anonymous ECGs which were subsequently reviewed by a senior cardiologist. Cardiologists could not identify the 12‐lead and single‐lead recordings that belonged to the same patient. QT intervals were manually determined in lead II according to the tangent method (Postema & Wilde, 2014). Lead II was chosen because this resulted in easily recognizable T waves. The Bazett's formula was used to correct for the heart rate (QTc). Other parameters registered included heart rate, RR interval, baseline rhythm (sinus, atrial fibrillation, atrial flutter, sinus tachycardia, and pacemaker rhythm), conduction delay (PR and QRS prolongation), and extrasystoles (premature atrial beats and premature ventricular beats).

QTc intervals between the Heartcheck device and the 12‐lead ECG were compared using the Bland–Altman method for analysis of measurement agreement. The mean and 95% confidence interval of the difference in QTc intervals between the two methods for each patient (i.e., limits of agreement) were calculated (12‐lead were reference values). QTc intervals of the single‐lead recordings were compared with those of the 12‐lead (reference group). Perfect agreement was defined as a limit of agreement of 10 ms or less between the two measurements. This choice was motivated by the fact that patients with a QTc interval of below 450–460 ms (males/females) are generally considered not critical but above this range are considered critical. This choice was recorded in the study protocol before data collection started. Rhythm abnormalities detected on both ECG recordings were compared. Statistical analysis was performed in SPSS version 25.

The sample size was chosen such that the observed limits of agreement could be claimed, with 95% confidence to be within the limits of perfect agreement. If there would be no bias of the single‐lead ECG compared to the 12‐lead ECG the true limit of agreement is 8.5 ms. When including 100 patients, the standard error in the limit of agreement is $\sqrt{\frac{3}{n}}≅0.17$ times the *SD* of the differences (Giavarina, 2015) and the limit of agreement would be 1.96 times the *SD* of the difference (assuming no bias). Thus, if there is no bias between the methods, a limit of agreement of 8.5 or less will then have 95% confidence interval that is within 10 ms.

## 3 RESULTS

**Figure 1:**
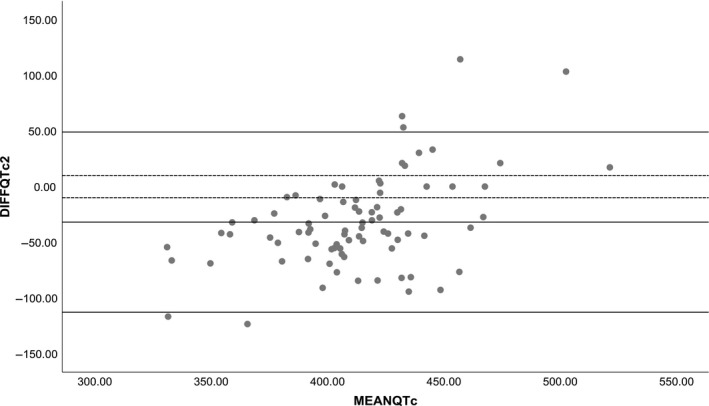
Comparison of manually determined QTc‐intervals for the 12‐lead and single‐lead ECG recordings using the Bland–Altman method for analysis of measurement agreement. The middle solid line represents the mean difference, the upper and lower solid line give the range in which 95% of the observed differences fall (“observed limits of agreement”), and the dotted lines the perfect limit of agreement (10 ms or less)

ECG records were obtained from 101 patients (50.5% female, mean age [*SD*] 61.1 [16.9] years). Overall, the Heartcheck was easy to use for measuring ECG recordings. Seventeen (16.8%) single‐lead ECG recordings were of insufficient quality to interpret QTc intervals. The mean QTc interval measured was 430.6 [*SD* ± 31.1] ms for the 12‐lead, and 396.7 [*SD* ± 47.5] ms for the single‐lead. The difference of the QTc intervals between the two measurements was substantially outside our definition of perfect agreement of 10 ms difference or less (mean difference −31.3 [*SD* ± 41.2] ms, Figure 1). Only 7 (6.9%) ECG recordings demonstrated perfect agreement.

Table 1 summarizes the variations in heart rate, conduction delays, and extrasystoles determined on both recordings. Almost all abnormalities were not consistently detected on both the 12‐lead and single‐lead ECG recordings. Abnormalities detected with the 12‐lead were found to be normal on the single‐lead recordings and vice versa.

**Table 1:** Clinical parameters determined on the 12‐lead and single‐lead ECG recordings and the number thereof that was identified with the single‐lead

	12‐lead *n* (%)	Single‐lead *n* (%)	Recorded on both the 12‐lead and single‐lead *n*
Number of patients with sufficient ECG quality	101 (100%)	84 (100%)	
Rhythm			
Sinus rhythm	87 (86.1)	75 (89.3)	72
Atrial fibrillation	12 (11.9)	8 (9.5)	6
Atrial flutter	2 (2.0)	–	–
Conduction delay			
PR prolongation	6 (5.9)	–	–
QRS prolongation (>120 ms)	13 (12.9)	7 (8.3)	5
Extrasystoles			
Premature atrial contractions	1 (1.0)	4 (4.5)	1
Premature ventricular contractions	5 (5.0)	5 (5.9)	2

## 4 DISCUSSION

This study shows that the Heartcheck single‐lead portable ECG device had inferior diagnostic accuracy to measure QTc intervals in cardiology patients compared with the gold‐standard 12‐lead ECG, when considering a limit of agreement 10 or less as perfect agreement. Numerous single‐lead ECG recordings contained artefacts that inhibited an adequate interpretation.

To our knowledge, only one study assessed the diagnostic accuracy of a single‐lead ECG device for QTc‐interval measurements and showed good agreement in healthy volunteers compared with a 1‐lead ECG (bias 4 ms *SD* 11 ms) but reasonable agreement in hospitalized patients on QTc‐prolonging drugs (bias 3 ms *SD* 43 ms) (Garabelli et al., 2016). Malone et al showed feasibility of measuring QTc intervals with a single‐lead portable ECG device (Alivecor) in the community setting (Malone, Gallo, Beck, & Clark, 2017); however, the manufacturer declares that the device does not have capability to detect QTc intervals. In our view, current (single‐lead) technology may provide limited possibilities to monitor patients using QTc‐prolonging drugs in clinical practice. We strongly recommend that diagnostic accuracy of such devices should be established prior to application in clinical practice. New innovations are needed to adequately record ECG’s without artefacts.

This study included a sufficiently large sample of patients to enable an assessment of the diagnostic accuracy of the portable ECG device compared with the 12‐lead ECG. One of the major limitations is that no information is provided on the agreement of devices regarding additional interval measurements. This is out of the scope of this study, which was powered for agreement between QTc intervals. For other ECG intervals there are adequate devices that can be used.

## 5 CONCLUSION

In comparison with a 12‐lead ECG device, the Heartcheck^TM^ single‐lead portable ECG device was insufficient to provide diagnostic accuracy to measure QTc intervals in cardiac patients.

## CONFLICT OF INTEREST

None.
